# Supplementary material for: Association between Microsatellite Instability Status and Peri-Operative Release of Circulating Tumour Cells in Colorectal Cancer
Source: Cells. 2020 Feb 12;9(2):425. doi: 10.3390/cells9020425 (PMC7072224; doi:10.3390/cells9020425)
Supplement: Supplementary file 1 [file cells-09-00425-s001.pdf]

Article

Supplementary Material

Table S1. Patient, MSI status, stage and CTC number at different time points.

Patient*	MSI Status	Stage	CTC Number			
			t1	t2	t3	t4
S1	MSS	I	8	5	2	1
S2	MSI-H	II	11	55	1	6
S3	MSS	II	11	12	7	11
S4	MSS	II	0	1	0	0
S5	MSS	I	5	4	2	12
S6	MSS	III	1	0	0	0
S8	MSS	III	0	1	0	6
S9	MSS	III	2	0	0	0
S10	MSI-H	III	29	44	38	57
S11	MSS	III	2	1	1	0
S12	MSI-H	II	10	49	8	11
S13	MSS	II	0	0	1	0
S14	MSS	I	0	0	0	0
S15	MSS	II	0	1	0	2
S16	MSS	IV	13	189	83	65
S18	MSI-H	III	0	3	2	1
S19	MSS	III	61	74	17	12

*Non- cancer patients were excluded from the Table
